# Effects of combined netupitant and palonosetron (NEPA), a cancer supportive care antiemetic, on the ECG of healthy subjects: an ICH E14 thorough QT trial

**DOI:** 10.1186/2193-1801-3-389

**Published:** 2014-07-29

**Authors:** Tulla Spinelli, Cecilia Moresino, Sybille Baumann, Wolfgang Timmer, Armin Schultz

**Affiliations:** Helsinn Healthcare SA, Via Pian Scairolo 9, 6912 Lugano/Pazzallo, Switzerland; CRS Clinical Research Services Mannheim GmbH, Grenadierstrasse 1, 68167 Mannheim, Germany

**Keywords:** NEPA, Chemotherapy-induced nausea and vomiting, Netupitant, Palonosetron, QTc, ECG

## Abstract

Chemotherapy-induced nausea and vomiting is ranked among the worst side effects of chemotherapy. NEPA is an oral fixed-dose combination antiemetic under development, consisting of netupitant 300 mg, a highly selective NK_1_ receptor antagonist (RA), and palonosetron 0.5 mg, a pharmacologically and clinically distinct 5-HT_3_ RA. Although palonosetron is not associated with relevant ECG effects, this study evaluated cardiovascular safety of netupitant in combination with palonosetron, as well as its tolerability.

This randomised, placebo- and positively controlled study in 197 subjects included 4 treatment groups: placebo, 200 mg netupitant + 0.5 mg palonosetron (NEPA_200/0.5_), 600 mg netupitant + 1.5 mg palonosetron (NEPA_600/1.5_, a supratherapeutic dose), and 400 mg moxifloxacin. Assessments included a 24-h baseline ECG recording, followed by a single dose of treatment and ECG measurements for 2 days.

Mean placebo-corrected time-averaged changes from baseline were similar in NEPA_200/0.5_ and NEPA_600/1.5_ groups primarily for individually heart rate-corrected QT interval (QTcI: +4.7 and +3.6 ms, respectively) and for heart rate (HR: –3.3 bpm and –3.0 bpm), PR interval (–0.4 ms and 0.2 ms), and QRS interval (1 ms and 0.5 ms). The time-matched analysis showed no upper confidence interval >10 ms, with no suggestion of a QTc effect by pharmacokinetic-pharmacodynamic modeling for parent/metabolites. Moxifloxacin showed the expected placebo-corrected change from baseline (+8.4 ms time average) and the expected profile to establish assay sensitivity. No new morphologic changes of clinical relevance were observed. Treatment-related adverse events were comparable among groups.

This study showed that NEPA treatments produced no significant effects on QTcI, HR, PR interval, QRS interval, and cardiac morphology relative to placebo, even at supratherapeutic doses.

## Background

Chemotherapy-induced nausea and vomiting (CINV) is a common and distressing consequence of cytotoxic chemotherapy. Acute CINV is described as CINV occurring in the first 24 hours after administration of chemotherapy, whereas delayed CINV begins 25 hours or more after chemotherapy initiation, and can last up to several days after chemotherapy is completed (Bloechl-Daum et al. 2006; Hesketh et al. 2003). CINV impacts patients’ quality of life and is a major reason for noncompletion or delay of the chemotherapy programme (Bloechl-Daum et al. 2006; Aapro et al. 2012; Cohen et al. 2007).

There are 2 major pathways known to be involved in CINV. The neurotransmitter serotonin (5-hydroxytryptamine or 5-HT) has been shown to be an important mediator of the acute phase, while the role of substance P is mainly related to the delayed phase of CINV (Hesketh et al. 2003; Rojas and Slusher 2012; Feyer and Jordan 2011; Rubenstein et al. 2006). Preclinical studies demonstrated that cisplatin causes increased levels in the peripheral circulation of both serotonin and substance P. The 5-HT_3_ receptor antagonists (RAs) are thought to inhibit the serotonin emetic pathway peripherally, while the neurokinin 1 (NK_1_) RAs are thought to act on the substance P-mediated signaling at the level of the central nervous system (Hesketh et al. 2003; Rojas and Slusher 2012; Feyer and Jordan 2011; Rubenstein et al. 2006).

International antiemetic guidelines recommend administering a 5-HT_3_ RA with an NK_1_ RA and a corticosteroid as part of the antiemetic regimen to prevent nausea and vomiting in patients who are at high risk to develop it (Basch et al. 2011; Gralla et al. 2013; Roila et al. 2010; National Comprehensive Cancer Network 2013). Nevertheless, CINV is still underestimated, particularly in the delayed phase and with regard to nausea (Bloechl-Daum et al. 2006; Cohen et al. 2007; Salsman et al. 2012; Roscoe et al. 2004). This represents an area of need that should be addressed by new and safe antiemetics.

NEPA is a new antiemetic under development that targets a dual antiemetic pathway with a single oral fixed-dose combination of netupitant 300 mg and palonosetron 0.5 mg to be administered prior to emetogenic chemotherapy. The phase II and III pivotal clinical studies demonstrating both the safety and high efficacy of this convenient single-day antiemetic have recently been published (Hesketh et al. 2014; Aapro et al. 2014; Gralla et al. 2014). Netupitant (2-(3,5-Bis-trifluoromethyl-phenyl)-N-methyl-N-[6-(4-methyl-piperazin-1-yl)-4-o-tolyl-pyridine-3-yl]-isobutyramide) is a new and selective NK_1_ RA showing a high receptor occupancy level at time to maximum plasma concentration (t_max_; more than 90%) and a long-lasting (up to 96 hours postdose) blockade of NK_1_ receptors in the human brain (Spinelli et al. 2014). Chronic administration of different daily doses of netupitant (50 mg, 100 mg, and 200 mg) for 8 weeks raised no safety issues in patients with an overactive bladder (Haab et al. 2014). Palonosetron ((3aS)-2-[(S)-1-Azabicyclo[2.2.2]oct-3-yl]-2,3,3a,4,5,6-hexahydro-1-oxo-1Hbenz[de]isoquinoline hydrochloride) is a 5-HT_3_ RA with a longer half-life and higher binding affinity that differs from traditional setrons both from a pharmacologic and clinical point of view (Reddy et al. 2006). In vitro and in vivo studies demonstrated that palonosetron uniquely: 1) exhibits allosteric binding to the 5-HT_3_ receptor, with positive cooperativity and persistent inhibition of receptor function; 2) triggers 5-HT_3_ receptor internalisation; and 3) inhibits substance P-mediated response through inhibition of the 5-HT_3_ and NK_1_ receptor cross-talk (Rojas and Slusher 2012). Several studies have shown that palonosetron, as a single agent or in combination with a corticosteroid, has a high tole rability profile and achieves superior efficacy in preventing CINV compared with the other 5-HT_3_ RAs (Aapro et al. 2006; Eisenberg et al. 2003; Gralla et al. 2003; Saito et al. 2009).

Preclinical data demonstrated that NEPA synergistically enhanced inhibition of the substance P response compared to either palonosetron or netupitant alone (Stathis et al. 2012). These data suggest that the NEPA combination represents an effective and convenient approach to prevent acute and delayed CINV with a single oral dose.

Cardiovascular disease represents one of the most common comorbidities in the growing population of cancer patients aged more than 65 years (Aapro et al. 2005). Cardiopathy can be preexisting or a consequence of the malignancy, and ECG changes can be an adverse event (AE) resulting from chemotherapy treatment. Several antineoplastic agents (especially anthracyclines) and some platinum compounds are associated with ECG alterations, including prolongation of the QT interval, development of ventricular late potentials, and various arrhythmias. It has been shown that the interaction of anthracyclines with the monoclonal antibody trastuzumab, which is quite common in breast cancer patients, can potentiate cardiotoxic effects (Bagnes et al. 2010). Cyclophosphamide treatment has been associated with 7% to 28% incidence of heart failure, while cisplatin has been associated with 8.5% of venous thromboembolism incidence (Vo and Nelson 2012). The use of 5-fluorouracil has been reported to be associated with ECG changes (eg, ST segment deviation and corrected QT interval [QTc] prolongation in nearly 68% of patients) and cardiotoxicity (eg, angina, supraventricular tachycardia, and myocardial infarction with an incidence of 1%–18%) (Sorrentino et al. 2012). Cardiotoxicity has also been reported after administration of taxanes (eg, brady- and tachyarrhythmia and other cardiac disturbances) (Bagnes et al. 2010; Yeh and Bickford 2009). The incidence of bradycardia associated with paclitaxel treatment ranges from <0.1% to 31% and the incidence of heart failure associated with docetaxel treatment ranges from <2.3% to 8% (Yeh and Bickford 2009). Both paclitaxel (<1%–5% incidence) and docetaxel (1.7% incidence) have been associated with myocardial ischaemia (Yeh and Bickford 2009).

Not only chemotherapeutic agents, but also newer targeted agents have been shown to lead to cardiotoxicity (Bagnes et al. 2010; Hedhli and Russell 2011). Multikinase-targeted drugs (eg, sorafenib, sunitinib, imatinib, and dasatinib) are associated with various cardiac effects, such as hypertension, congestive heart failure, and QTc prolongation (Bagnes et al. 2010; Hedhli and Russell 2011). The use of histone deacetylase inhibitors has also been associated with QTc prolongation (Bagnes et al. 2010; Hedhli and Russell 2011). Administration of anti-vascular endothelial growth factor agents (eg, bevacizumab and aflibercept) can lead to hypertension, arrhythmia, and thromboembolic effects, such as myocardial infarction (Bagnes et al. 2010; Hedhli and Russell 2011).

The current double-blind, randomised, parallel-group study evaluated whether the combined administration of different doses of netupitant + palonosetron prolongs the individually heart rate-corrected QT interval (QTcI) more than placebo. Safety and tolerability of the combination therapy, as well as pharmacokinetic (PK) data, were evaluated.

## Methods

### Study design and treatment

This was a phase I, randomised, double-blind (except for the use of moxifloxacin), double-dummy, parallel-group, placebo- and open-label positively controlled study (EudraCT: 2007-004365-17). The study was approved by appropriate ethics committees and was conducted in accordance with the Declaration of Helsinki, the German Drug Law, and the German Good Clinical Practice decree. All subjects gave written informed consent to participate in the trial. The primary objective of this study was to assess whether the combined administration of different doses of netupitant + palonosetron prolongs QTcI more than placebo. Secondary objectives were to evaluate the safety and tolerability of NEPA combinations and assess the PK of netupitant, palonosetron, and their metabolites.

The trial design followed the provisions of ICH Guideline E14 for a “thorough QT/QTc study” (US Department of Health and Human Services 2005). The study consisted of an ambulant screening phase (days –21 to –3), a pre-check period (day –2 and –1), a treatment period (days 1–3), and an ambulant final check 14 to 21 days after discharge from the study center on day 3. Subjects were hospitalised from the evening of day –2 until day 3. Subjects were randomly assigned to one of the following single-dose treatment groups: placebo, 200 mg netupitant + 0.50 mg palonosetron (NEPA_200/0.5_), 600 mg netupitant + 1.50 mg palonosetron (NEPA_600/1.5_), and 400 mg moxifloxacin (Avelox®, Bayer Healthcare; positive control). A 24-hour baseline ECG was followed by a single dose of treatment on day 1, after which subjects had ECG and PK measurements up to 48 hours postdose.

### Subjects

Two hundred healthy subjects (at least 92 of each gender) aged 18 to 45 years were included if they had a body mass index of 19 to <29 kg/m^2^, were nonsmokers (or refrained from smoking or taking other nicotine-containing products for 3 months prior to dosing), and had normal blood pressure (55–89/95–149 mmHg) and pulse rate (45–95 beats/minute). Subjects with any of the following were excluded: current use of oral contraceptives or hormones within 3 months prior to dosing; pregnant or breastfeeding; use of prescribed or over-the-counter medication within 14 days of dosing; any active physical disease (acute or chronic); gastrointestinal complaints within 7 days of dosing; febrile or infectious illness within 7 days of dosing; any cardiovascular condition; any abnormal ECG interval or changes in ECG that might interfere with measurement of QT interval; relevant drug hypersensitivity (specifically against moxifloxacin); known contraindication to NK_1_ RAs, 5-HT_3_ RAs, or fluoroquinolones; positive test for hepatitis B virus, hemoglobin C, or human immunodeficiency virus; or any other reason deemed unsuitable in the opinion of the investigator.

### Pharmacodynamics

ECGs were obtained using a continuous 12-lead digital Holter recorder (Mortara H-12; Milwaukee, WI) on day –1 (baseline) and on days 1 and 2. ECGs to be used in the analysis were selected at the following predetermined time points: day –1 at –23, –22, –20, –19, –18, –17, –16, –14, –12, –10, –8, –6, and –0.5 hours; days 1 and 2 at 1, 2, 4, 5, 6, 7, 8, 10, 12, 14, 16, 18, 23.5, 30, 36, 42, and 47.5 hours. Four ECGs were obtained at each time point. Three kinds of QTc were calculated. The primary ECG endpoint was QTcI, calculated as QTcI = QT/(RR)^slope^, where the slope was determined for each subject by linear regression analysis on the baseline ECGs. Secondary ECG variables included frequency correction performed using the Fridericia formula (QTcF) and frequency correction performed using Bazett formula (QTcB). Additionally, the following parameters were evaluated: uncorrected QT interval, heart rate (HR), PR interval, QRS interval, and change in ECG morphologic patterns.

### Pharmacokinetics

The area under the plasma concentration-time curve data from administration until the last sampling point (AUC_0-t_), maximum plasma concentration (C_max_), and t_max_ were determined for netupitant (and its metabolites M1, M2, and M3) and palonosetron (and its metabolites M4 and M9), if data permitted. AUC_0-t_ was calculated by the linear trapezoidal formula, C_max_ was defined as the highest observed plasma concentration of the measured concentration-time profile, and t_max_ was set as the time after administration at which C_max_ occurred.

A simultaneous, validated, internally standardised liquid chromatography-tandem mass spectrometry (LC-MS/MS) method with electrospray ionisation in the positive mode was used for the analysis of netupitant and its metabolites M1, M2, and M3 and palonosetron and its metabolites M4 and M9. The analytics methods used for determination of netupitant and palonosetron and their metabolites are validated and approved by the US Food and Drug Administration and European Medicines Agency guidelines and were performed accordingly.

### Safety and tolerability parameters

Safety and tolerability parameters included physical examination, vital signs, body temperature, body weight, ECG recording, and laboratory examinations (clinical chemistry, haematology, urinalysis, serology, drug screen and alcohol breath test, and pregnancy test). AEs were ascertained and rated by the investigators. Overall tolerability was assessed by the investigator at the end of the study.

### Statistics methods

Biometric and PK evaluation were carried out using SAS® (Version 9.1). Descriptive statistics were used to summarise demographic data and ECG variables at each time point. For the QTc analysis, 2-sided 90% confidence intervals (CIs) based on the intersection–union test were calculated for each matched time (time-matched analysis). If the upper limit of the 2-sided 90% CI for the study treatment versus placebo did not exceed 10 milliseconds (ms) at any time point, it was concluded that this dose did not prolong the QTc interval to a clinically significant degree. To establish assay sensitivity, at least 1 time point with a mean difference of moxifloxacin and placebo >5 ms had to be observed.

In addition to the time-matched analysis, the time-averaged analysis was calculated. For each subject and for the ECG parameters QTc (I, B, F), HR, PR, QRS, and QT, the mean of all baseline ECGs was calculated as the time-averaged baseline value, and the mean of all postdose ECGs was calculated as the time-averaged postdose value. The time-averaged change from baseline was calculated from both values. The placebo-corrected time-averaged change from baseline was calculated by subtracting the mean placebo baseline-corrected values from the mean time-averaged change from baseline of the other treatment groups.

The PK/pharmacodynamics (PD) analysis explored the relationship between the placebo-adjusted QTcI change from baseline and plasma concentrations of netupitant and palonosetron. A linear mixed-effects modeling approach was adopted in which this PK/PD relationship was a fixed effect with subject included as a random effect (ΔΔQTcI = α + β ∗ [plasma concentration] + γ ∗ [subject effect]). This model was used to estimate the population slope and the standard error (SE) of the slope. A linear relationship was declared if the p-value of the slope was <0.05. The mean maximum effect (C_max_ ∗ β) and the upper 1-sided 95% CI (C_max_ ∗ β + [1.65 ∗ SE β ∗ C_max_]) were calculated.

A sample size of 50 subjects per group was expected to provide at least 80% power to show for the comparison of netupitant/palonosetron to placebo that the upper limit of the 90% CI falls below 10 ms. The sample size was calculated based on an assumed standard deviation of 11 ms. The true difference between time-matched changes from baseline QTcI of netupitant/palonosetron and placebo groups was selected as 3 ms.

## Results

### Disposition and baseline characteristics

Two hundred subjects (106 males and 94 females) were enrolled in the study. Five women withdrew consent and discontinued the study. Three of them withdrew their consent before treatment administration and were therefore not included in any analysis. One subject in the NEPA_200/0.5_ group did not show netupitant plasma levels and was therefore excluded from the PD analyses. One hundred ninety-five subjects completed the study. The baseline characteristics of the study population were comparable between treatment groups and are shown in Table 1.

**Table 1:** **Baseline characteristics**

	Placebo (N = 50)	NEPA _200/0.5_ (N = 49)	NEPA _600/1.5_ (N = 49)	Moxifloxacin (N = 49)
Age, years				
Mean (SD)	34.7 (7.12)	33.6 (6.86)	32.8 (8.36)	34.7 (7.98)
Min–max	19–45	21–44	19–45	19–45
Gender, n (%)				
Male	26 (52)	27 (55)	27 (55)	26 (53)
Female	24 (48)	22 (45)	22 (45)	23 (47)
BMI, kg/m^2^				
Mean (SD)	24.45 (2.486)	24.27 (2.608)	24.51 (2.516)	24.89 (2.674)
Min–max	19.0–28.9	19.2–28.5	19.4–28.9	19.4–29.0

BMI, body mass index; SD, standard deviation.

NEPA_200/0.5_: 200 mg netupitant (50 mg + 150 mg) + 0.50 mg palonosetron (1 × 0.50 mg).

NEPA_600/1.5_: 600 mg netupitant (4 × 150 mg) + 1.50 mg palonosetron (3 × 0.50 mg).

Percentages are based on N.

### Pharmacodynamics

One hundred ninety-six subjects were included in the PD analyses. Because heart rate inversely affects QT duration, the assessment of cardiac repolarisation was based on QTcI. The QTcI mean time-averaged placebo-corrected change from baseline for NEPA_200/0.5_ and NEPA_600/1.5_ was +4.7 and +3.6 ms, respectively (Table 2). For QTcB, values were –0.9 and –0.5 ms for NEPA_200/0.5_ and NEPA_600/1.5_ groups, respectively. For the time-averaged analysis, the mean placebo-corrected change from baseline for heart rate was similar for the NEPA_200/0.5_ and NEPA_600/1.5_ groups (–3.3 and –3.0 bpm, respectively; Table 2). Mean placebo-corrected change from baseline for PR and QRS durations was similar for the NEPA_200/0.5_ (PR: –0.4 ms; QRS: 1.0 ms) and NEPA_600/1.5_ (PR: 0.2 ms; QRS: 0.5 ms) groups, and was not considered clinically relevant (Table 2).

**Table 2:** **Time-averaged analysis: mean changes from baseline**

Endpoint	Parameter	Placebo (N = 50)	NEPA _200/0.5_ (N = 48)		NEPA _600/1.5_ (N = 49)		Moxifloxacin (N = 49)	
		Change from baseline	Change from baseline	Placebo-corrected change from baseline	Change from baseline	Placebo-corrected change from baseline	Change from baseline	Placebo-corrected change from baseline
Primary	QTcI, ms	–2.1	2.6	4.7	1.5	3.6	6.3	8.4
	(Min;max)	(−8.3; 4.8)	(−5.8; 30.6)		(−7.3; 14.3)		(−2.6; 15.3)	
Secondary	HR, bpm	–0.7	–4.0	–3.3	–3.7	–3.0	–0.4	0.3
	PR, ms	1.1	0.7	–0.4	1.3	0.2	–0.2	–1.3
	QRS, ms	–0.3	0.7	1.0	0.2	0.5	0.0	0.3
	QT, ms	–0.4	9.3	9.7	8.6	9.0	6.9	7.3
	QTcF, ms	–2.1	0.5	2.6	0.5	2.6	6.0	8.1
	QTcB, ms	–3.0	–3.9	–0.9	–3.5	–0.5	5.5	8.5

bpm, beats per minute; min, minimum; max, maximum; HR, heart rate; ms, milliseconds; QTcB, Bazett correction; QTcF, Fridericia correction; QTcI, individually heart rate-corrected QT interval.

NEPA_200/0.5_: 200 mg netupitant (50 mg + 150 mg) + 0.50 mg palonosetron (1 × 0.50 mg).

NEPA _600/1.5_: 600 mg netupitant (4 × 150 mg) + 1.50 mg palonosetron (3 × 0.50 mg).

These data showed no signs for an effect of different doses of netupitant + palonosetron on QTc (Table 2). In the moxifloxacin-treated group, the placebo-corrected mean change from baseline for QTcI and QTcB values was +8.4 and +8.5 ms, respectively (expected 5–10 ms), indicating assay sensitivity was reached (Table 2). In the placebo group the QTcI and QTcB mean changes from baseline were –2.1 and –3.0 ms, respectively, showing that background QTc was controlled (Table 2).

**Figure 1:**
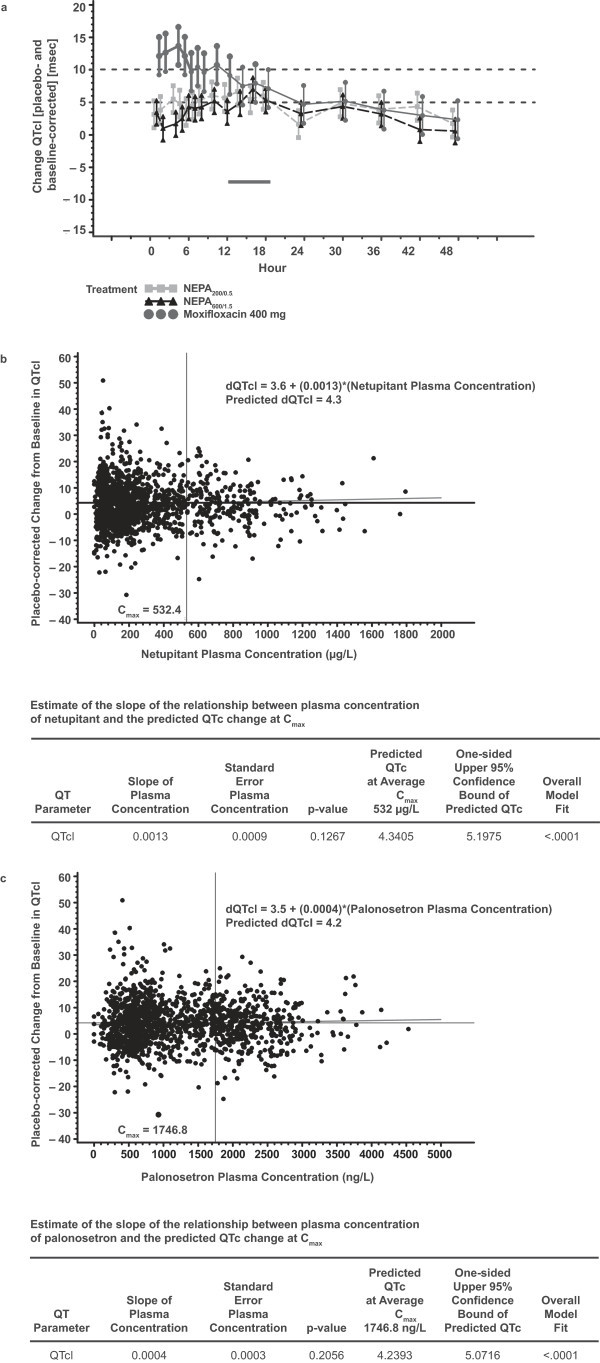
**Placebo- and baseline-corrected change in QTcI. (a)** Placebo- and baseline-corrected change in QTcI for each time point in each treatment group. **(b)** Pharmacokinetic/pharmacodynamics relationship: placebo- and baseline-corrected change in QTcI versus netupitant plasma concentrations and **(c)** versus palonosetron plasma concentrations.

A time-matched analysis was conducted as recommended by ICH E14 with placebo- and baseline-corrected QTcI data for NEPA and moxifloxacin dose groups. The analysis for QTcI revealed that the moxifloxacin group met the assay sensitivity criteria with 12 time points above a mean of 5 ms (Table 3, Figure 1a). Results showed that the NEPA groups did not exceed the upper CI of 10 ms at any of the time points (Table 3, Figure 1a). Therefore, the combination of different doses of netupitant and palonosetron did not prolong the QTc interval to a clinically significant degree.

**Table 3:** **Time-matched analysis: placebo and baseline corrected QTcI data for NEPA and moxifloxacin dose groups**

Time, h	NEPA _200/0.5_		NEPA _600/1.5_		Moxifloxacin	
	Estimate ^1^	Upper bound ^2^	Estimate ^1^	Upper bound ^2^	Estimate ^1^	Upper bound ^2^
1	3.2	5.2	3.5	5.3	12.2	15.1
2	3.8	5.9	1.0	2.8	12.7	15.6
4	5.5	7.6	1.7	3.5	13.7	16.6
5	4.8	6.9	2.5	4.3	12.2	15.1
6	3.5	5.6	4.3	6.1	9.7	12.6
7	4.5	6.5	4.1	5.9	10.5	13.4
8	5.3	7.4	4.3	6.1	9.6	12.6
10	6.1	8.1	5.2	7.0	10.8	13.7
12	5.7	7.8	3.6	5.4	9.2	12.1
14	6.7	8.7	4.9	6.7	7.5	10.4
16	5.5	7.6	7.0	8.8	8.0	10.9
18	6.0	8.1	5.3	7.1	7.1	10.0
23.5	1.7	3.7	3.3	5.1	4.7	7.6
30	4.9	7.0	4.4	6.2	5.2	8.1
36	3.9	6.0	3.2	5.0	3.8	6.7
42	4.3	6.4	0.8	2.6	3.0	5.9
47.5	1.8	3.8	0.6	2.4	2.3	5.3
Time average	4.6	5.7	3.4	4.5	8.4	9.5

ANOVA, analysis of variance; QTcI, individually heart rate-corrected QT interval.

NEPA_200/0.5_: 200 mg netupitant (50 mg + 150 mg) + 0.50 mg palonosetron (1 × 0.50 mg).

NEPA_600/1.5_: 600 mg netupitant (4 × 150 mg) + 1.50 mg palonosetron (3 × 0.50 mg).

^1^Mixed-model ANOVA is fit for placebo-corrected change from baseline and includes terms for treatment, gender, time, and a time-by-treatment interaction.

^2^Upper bound, upper 1-sided 95% ANOVA model-based confidence limit.

No significant gender effect was observed. Morphologic changes were not considered clinically significant in any dose group and there were no imbalances in the NEPA_200/0.5_ and NEPA_600/1.5_ groups.

### Pharmacokinetics

PK parameters were assessed for netupitant and its metabolites M1, M2, and M3 and palonosetron and its metabolites M9 and M4.

A dose-proportional increase of the geometric mean AUC_0-t_ was observed for netupitant (4079 h ∗ μg/L at 200 mg to 12213 h ∗ μg/L at 600 mg) and palonosetron (22641 h ∗ ng/L at 0.5 mg to 67918 h ∗ ng/L at 1.5 mg). Similarly, C_max_ increased with dose for both netupitant (219 μg/L to 648 μg/L) and palonosetron (822 ng/L to 2588 ng/L; Table 4). The intersubject PK variability was higher for netupitant than for palonosetron. The intersubject variability of netupitant for AUC_0-t_ and C_max_ was 42% and 48% at 200 mg, and 47% and 56% at 600 mg. For palonosetron, the intersubject variability for AUC_0-t_ and C_max_ varied from 20% to 29%. The pharmacokinetics of netupitant and palonosetron, including their metabolites, confirm that the ECG time points were adequately chosen, since the C_max_ of all analytes lie within the ECG measurement time points.

**Table 4:** **Descriptive statistics of PK parameters for netupitant and palonosetron**

Netupitant	AUC _0-t_ (h ∗ μg/L) Geo. Mean (Geo. SD)	C _max_ (μg/L) Geo. Mean (Geo. SD)	t _max_ (h) Geo. Mean (Geo. SD)
NEPA_200/0.5_			
Parent	4079 (1.712)	218.7 (1.833)	5.218 (1.589)
M1	832.5 (1.719)	23.55 (1.594)	14.54 (1.668)
M2	827.3 (1.722)	96.94 (1.842)	3.580 (1.342)
M3	1348 (1.642)	46.73 (1.559)	10.70 (1.490)
NEPA_600/1.5_			
Parent	12213 (1.925)	647.7 (2.205)	6.104 (1.474)
M1	2007 (1.766)	53.71 (1.763)	17.82 (1.948)
M2	2155 (1.908)	227.8 (2.024)	4.131 (1.208)
M3	2981 (1.707)	101.5 (1.822)	11.51 (1.555)
**Palonosetron**	**AUC**_**0-t**_ **(h** ∗ **ng/L) Geo. Mean (Geo. SD)**	**C**_**max**_ **(ng/L) Geo. Mean (Geo. SD)**	**t**_**max**_ **(h) Geo. Mean (Geo. SD)**
NEPA_200/0.5_			
Parent	22641 (1.241)	821.6 (1.277)	5.464 (1.437)
M4	541.7 (2.916)	72.72 (1.379)	3.675 (1.764)
M9	416.2 (2.158)	105.0 (1.383)	1.746 (1.348)
NEPA_600/1.5_			
Parent	67918 (1.210)	2588 (1.239)	4.229 (1.693)
M4	5571 (1.348)	231.0 (1.330)	3.388 (1.734)
M9	3973 (1.468)	348.9 (1.313)	1.781 (1.535)

AUC_0-t_, area under the plasma concentration-time curve data from administration until the last sampling point; C_max_, maximum plasma concentration; Geo., geometric; PK, pharmacokinetic; SD, standard deviation; t_max_, time to C_max_.

NEPA_200/0.5_: 200 mg netupitant (50 mg + 150 mg) + 0.50 mg palonosetron (1 × 0.50 mg).

NEPA_600/1.5_: 600 mg netupitant (4 × 150 mg) + 1.50 mg palonosetron (3 × 0.50 mg).

### Pharmacodynamics/pharmacokinetics relationship

The relationship between placebo- and baseline-corrected QTcI duration and plasma concentration from paired samples taken in both dose groups for netupitant (Figure 1b) and palonosetron (Figure 1c) was evaluated. There were no indications that exposure to netupitant or palonosetron (parent or M1, M2, or M3 metabolites for netupitant; M9 or M4 metabolite for palonosetron) induced changes in QTc parameters. All estimated slopes were close to 0 and no upper CI approached 10 ms.

### Safety and tolerability

During the study, a total of 60 AEs were reported by 49 subjects. AEs were reported for 12 (24.0%) subjects in the placebo, 10 (20.4%) in the NEPA_200/0.5_, 17 (34.7%) in the NEPA_600/1.5_, and 10 (20.4%) in the moxifloxacin groups. Possibly or probably treatment-related AEs (TRAEs) were reported for 9 (18.0%) placebo subjects and 8 (16.3%), 10 (20.4%), and 8 (16.3%) subjects in the NEPA_200/0.5_, NEPA_600/1.5_, and moxifloxacin groups, respectively (Table 5). The incidence of TRAEs in the NEPA_200/0.5_ group was comparable to that of the placebo and moxifloxacin groups, although the frequency of TRAEs was slightly higher with NEPA_600/1.5_. With NEPA_200/0.5_ treatment, most commonly reported TRAEs were constipation (3 subjects), followed by upper abdominal pain (2 subjects), and headache (2 subjects). In the NEPA_600/1.5_ group, headache (5 subjects) was most commonly reported, followed by constipation (2 subjects). Subjects treated with moxifloxacin reported mainly dizziness (3 subjects) and headache (2 subjects).

**Table 5:** **Possibly or probably drug-related adverse events**

Adverse event	Placebo	NEPA _200/0.5_	NEPA _600/1.5_	Moxifloxacin
Number of subjects (%)	(N = 50)	(N = 49)	(N = 49)	(N = 49)
Cardiac disorders				
Palpitations	0	0	1 (2.0)	0
Gastrointestinal disorders				
Abdominal pain upper	0	2 (4.1)	0	0
Constipation	0	3 (6.1)	2 (4.1)	0
Dry mouth	1 (2.0)	0	0	0
Dyspepsia	1 (2.0)	0	0	0
Flatulence	1 (2.0)	0	0	0
Nausea	0	0	0	1 (2.0)
General disorders and administration-site conditions				
Fatigue	0	1 (2.0)	0	1 (2.0)
Thirst	0	0	0	1 (2.0)
Musculoskeletal and connective tissue disorders				
Muscle twitching	1 (2.0)	0	0	0
Pain in extremity	0	0	1 (2.0)	0
Sensation of heaviness	1 (2.0)	0	0	0
Nervous system disorders				
Dizziness	1 (2.0)	0	1 (2.0)	3 (6.1)
Headache	5 (10.0)	2 (4.1)	5 (10.2)	2 (4.1)
Somnolence	0	0	1 (2.0)	0
Psychiatric disorders				
Anxiety	0	0	1 (2.0)	0
Euphoric mood	0	0	1 (2.0)	0
Total	9 (18.0)	8 (16.3)	10 (20.4)	8 (16.3)

NEPA_200/0.5_: 200 mg netupitant (50 mg + 150 mg) + 0.50 mg palonosetron (1 × 0.50 mg).

NEPA_600/1.5_: 600 mg netupitant (4 × 150 mg) + 1.50 mg palonosetron (3 × 0.50 mg).

Percentages are based on N.

No deaths occurred during the study. One subject had a serious AE (injury) caused by a staircase accident and recovered by the end of the study. The investigator assessed the event as unlikely related to study drug. No clinically significant laboratory changes were observed and there were no AEs associated with laboratory changes. No abnormalities were detected in vital signs or ECG. The overall tolerability was assessed as good in 193 subjects and as satisfactory in 4 subjects.

## Discussion

NEPA is an oral fixed-dose combination of netupitant 300 mg and palonosetron 0.5 mg targeting dual antiemetic pathways mediated by serotonin and substance P with a single administration on the day of chemotherapy. Since the incidence of cancer patients with cardiovascular-related diseases is growing, especially in the elderly population, the cardiac safety profile of drugs used for supportive care should be investigated.

As recommended by international antiemetic guidelines, especially in patients undergoing highly emetogenic chemotherapy, an NK_1_ RA, a 5-HT_3_ RA, and a corticosteroid are commonly coadministered to prevent CINV (Basch et al. 2011; Gralla et al. 2013; Roila et al. 2010; National Comprehensive Cancer Network 2013). Aprepitant and its prodrug fosaprepitant are the only NK_1_ RAs currently available and used in clinical practice. A recent study investigating the cardiovascular safety of 200 mg fosaprepitant reported no clinically relevant effect on QTc interval at any time after infusion (including aprepitant t_max_) (Marbury et al. 2009; Emend (aprepitant) Prescribing Information 2006). The cardiovascular safety of another NK_1_ RA whose development was recently interrupted, casopitant, was evaluated and similarly showed no evidence of QTc prolongation after a 3-day oral regimen (Johnson et al. 2010).

Although 5-HT_3_ RAs such as dolasetron, ondansetron, and granisetron are perceived as safe by the medical community, cardiovascular issues have been reported. Ondansetron 4 mg has been associated with a statistically significant increased prolongation of QTc at various time points following administration in healthy subjects or surgical patients without additional risk factors for QTc prolongation (Charbit et al. 2005, 2008; Benedict et al. 1996; Zofran (ondansetron hydrochloride) Prescribing Information 2011). Although an adequate QT assessment was not conducted, QT prolongation has been associated with the use of granisetron, as shown in a study by Alidoosti et al. reporting prolongation of PR and QTc, and a decrease in HR in cancer patients (Alidoosti et al. 2009; Kytril (granisetron hydrochloride) Prescribing Information 2011). Dolasetron treatment resulted in dose-related increases in HR, PR intervals, and QRS intervals and a statistically significant increase in QTc interval (Benedict et al. 1996). Based on clinical evidence, dolasetron and the highest dose of ondansetron (32 mg) are no longer indicated for CINV prevention due to cardiovascular safety concerns. Contrary to these findings, recent studies, including a formal ICH E14 thorough QT trial, have reported that palonosetron does not prolong the QT interval and does not cause any significant acute change in repolarisation (QTc, corrected QT dispersion [QTcd]) or transmural dispersion indices (TpTe, TpTed, and TpTe/QT) (Dogan et al. 2012; Gonullu et al. 2012; Morganroth et al. 2007). The thorough QT/QTc (ICH E14) study in healthy subjects also demonstrated that palonosetron has no effect on the QT interval (QT/QTc) over the 0.25- to 2.25-mg range of exposure (Morganroth et al. 2007; Aloxi (palonosetron hydrochloride) Prescribing Information 2009). Yavas et al. demonstrated no acute effects of palonosetron on heart rate or blood pressure (Yavas et al. 2012).

This thorough ECG trial enrolled 200 healthy subjects to assess the impact of different doses of netupitant + palonosetron on cardiac repolarisation. The supratherapeutic dose of NEPA (NEPA_600/1.5_) was used to mimic exposure in healthy subjects that may occur in the target population under the worst circumstances (eg, concomitant liver disease, presence of heart disease, taking more than the prescribed clinical dose, etc.).

The present data demonstrate that different doses of NEPA have no significant effects on QTcl, HR, PR interval duration, QRS interval duration, or cardiac morphology compared with placebo. The validity of this trial was demonstrated by the fact that the moxifloxacin-positive control group showed the expected change in QTc duration to establish assay sensitivity. The placebo group’s change from baseline was within 3.0 ms, indicating that spontaneous factors for QTc change are well controlled. A dose-proportional increase in AUC_0-t_ and C_max_ was observed for both netupitant and palonosetron. Administration of different doses of NEPA was safe and well tolerated in healthy subjects. Most commonly reported TRAEs with NEPA groups were constipation, abdominal pain, and headache. Although TRAEs were slightly more frequent with NEPA_600/1.5_, the dose combination was safe with an acceptable tolerability profile.

This trial was performed in healthy subjects (18–45 years) to eliminate variables that are known to change ECG parameters, such as concomitant drugs and diseases. This study did not assess the cardiac safety of NEPA in cancer patients. However, a phase III study testing NEPA in cancer patients (without any serious cardiovascular disease history or predisposition to cardiac conduction abnormalities) receiving chemotherapy showed that there were no cardiac safety concerns for NEPA based on cardiac AEs and ECGs (Hesketh et al. 2014; Aapro et al. 2014; Gralla et al. 2014). Therefore, both in cancer patients and in healthy volunteers NEPA did not show any increased risk in cardiac safety profile.

In conclusion, in this thorough QT trial, different NEPA combinations showed no ECG effects, which should predict a lack of cardiac safety concerns in clinical practice. Treatments were well tolerated.

## Abbreviations

5-HT: 5-hydroxytryptamine
AE: Adverse event
AUC_0-t_: Area under the plasma concentration-time curve from administration until the last sampling point
CINV: Chemotherapy-induced nausea and vomiting
CIs: Confidence intervals
C_max_: Maximum plasma concentration
C_max_ ∗ β: Mean maximum effect
HR: Heart rate
LC-MS/MS: Liquid chromatography-tandem mass spectrometry
NEPA: Netupitant and palonosetron
NEPA_200/0.5_: 200 mg netupitant + 0.5 mg palonosetron
NEPA_600/1.5_: 600 mg netupitant + 1.5 mg palonosetron
NK_1_: Neurokinin 1
PD: Pharmacodynamics
PK: Pharmacokinetic
QTc: Corrected QT interval
QTcB: Frequency correction performed using Bazett formula
QTcd: Corrected QT dispersion
QTcF: Frequency correction performed using Fridericia formula
QTcI: Individually heart rate-corrected QT interval
RA: Receptor antagonist
SE: Standard error
t_max_: Maximum plasma concentration
TRAEs: Treatment-related adverse events.
